# Prevalence of dysmenorrhea and associated factors and its effect on daily academic activities among female undergraduate students of Hawassa University College of Medicine and Health Sciences, Hawassa, Ethiopia

**DOI:** 10.3389/frph.2023.1244540

**Published:** 2023-12-15

**Authors:** Wondu Belayneh, Zerai Kassaye, Temesgen Arusi, Netsanet Abera, Admassu Hantalo, Biruk Melkamu, Muluken Gutulo

**Affiliations:** ^1^ Department of Gynecology and Obstetrics, Hawassa University College of Medicine and Health Sciences, Hawassa, Ethiopia; ^2^ Obstetrics and Gynecology, Hawassa University College of Medicine and Health Sciences, Hawassa, Ethiopia; ^3^ Obstetrics and Gynecology, Wolkite University College of Medicine and Health Sciences, Wolkite, Ethiopia; ^4^ Department of Public Health, Hawassa University College of Medicine and Health Sciences, Hawassa, Ethiopia; ^5^ Department of Medical Laboratory Sciences, Wolkite University College of Medicine and Health Sciences, Wolkite, Ethiopia; ^6^ Wachamo University College of Medicine and Health Sciences, Hosanna, Ethiopia; ^7^ CDC Coordinator, Wolaita Health Department, Wolaita Sodo, Ethiopia

**Keywords:** dysmenorrhea, academic activities, family history, Hawassa University, depression

## Abstract

**Background:**

Dysmenorrhea is pain during menstrual flow and is the most common gynecologic complaint in reproductive-age girls. The severity of dysmenorrhea ranges from mild to severe pain during menstruation, which affects their academic activities.

**Objective:**

To assess the prevalence and associated risk factors of dysmenorrhea and its effects on academic activities among Hawassa University students from April 1–30/2021.

**Methods:**

A cross-sectional study was conducted and a systematic random sampling technique was used to select 348 study participants. Standardized self-administered questionnaires were used to obtain relevant data. The severity of pain was assessed using a verbal multidimensional scoring system and Numerical Pain Rating Scale (NPRS) methods. The collected data was entered into Epi info version 7 and exported to SPSS version 21 for analysis and then descriptive statistics and logistic regression analysis were performed.

**Results:**

The prevalence of dysmenorrhea was 80% (277). Of those affected by dysmenorrhea, using the verbal multidimensional scoring system, 47.6% (132) were found to have mild pain, 39.7% (110) had moderate pain, and 12.6% (35) had severe pain. However, using the Numeric Pain Rating Scale (NPRS), 21.7% (60) were found to have mild pain, 33.2% (92) had moderate pain, 37.5% (104) had severe pain, and 7.6% (21) had very severe pain. It was found to have a negative effect on academic activity, such as loss of concentration in class (*p* = 0.00), disruption of study time (*p* = 0.00), sleep disturbances (*p* = 0.00), issues in personal relationships (*p* = 0.00), and absenteeism (*p* = 0.00). Associated factors included being sexually active, having a family history of dysmenorrhea, and the presence of depression.

**Conclusion:**

Dysmenorrhea is prevalent in this study and has a significant impact on academic activities. Family history of dysmenorrhea, being sexually active, and the presence of depression are associated factors.

## 1. Introduction

Dysmenorrhea is lower abdominal pain during menstrual flow. It can be classified as primary or secondary dysmenorrhea based on the pathology. Primary dysmenorrhea is pain during menstrual flow without an identified cause. Secondary dysmenorrhea is pain during menstrual flow with an identified cause. The pain starts during the first 24–36 h of menstruation and lasts 2–3 days post menses. Primary dysmenorrhea is usually encountered during the earlier years of menstrual flow. The pathophysiology of pain is associated with increased prostaglandin production (PGF2α and PGE_2_) and abnormal uterine sensitivity to prostaglandins, which is a widely accepted theory. Increased production of prostaglandin in the face of increased sensitivity of uterine muscle to prostaglandin results in myometrium contractility, which exposes the uterus to hypoxia and ischemia in the uterus, which ultimately results in pain (1–6).

Since the reporting of pain is influenced by different sociocultural factors, the prevalence has wide variation across the globe. According to recent systemic reviews and meta-analysis, the pooled prevalence of primary dysmenorrhea ranges between 60% and 73% (7–10). However, other published reports indicate that the prevalence ranges from 16.8% to 95% (1, 2, 11–13). The Ethiopian prevalence is 64.7%−85.4% (14–16). The severity of dysmenorrhea varies across different reproductive age groups and countries. This disparity is possible because dysmenorrhea varies according to the age of women included in the study samples (17).

There are multiple factors associated with dysmenorrhea. Factors like age, alcohol use, family history, low BMI, the extent of menstrual flow, anxiety, chronic pelvic pain, and duration of flow are associated with dysmenorrhea (7, 8, 10, 18–22).

Dysmenorrhea has a significant impact on quality of life, academic performance, and personal relationships. This impact depends on the extent of the pain experienced. The effects on academic performance were found to mainly include study time, concentration, participation in group activities, examination performance, and class attendance. Social withdrawal, poor personal activities, sleep disturbances, and limited daily activities are also among the effects (1, 14, 15, 21, 23).

In Ethiopia, despite the negative sociocultural belief in women's empowerment programs, there has been significant progress in their participation in organizational and political leadership, the economy, and enrollment in higher education since the adoption of their empowerment plan in the past two decades. Women have participated in a significant number of Ethiopian political and economic activities in recent years. For example, women made up 50% of the Ethiopian cabinet in 2021, the country's president is a woman, and 35.7% of university students are female. Thus, equal involvement in social, intellectual, cultural, political, and economic concerns will be difficult to attain if there are obstacles that negatively affect young women's academic performance in universities (23–25).

Considering the valueless role of women in the country, these young female university students are of productive age and will become leaders of society and represent hope for the country and the community. Any problem that affects this age group will have a tremendous economic and psychological burden on the community and country. Furthermore, there is limited evidence on the prevalence, associated factors, and effects of dysmenorrhea on academic activities among female university students; thus, this study aims to identify these factors.

## 2. Methods

### 2.1. Study area and source population

An institution-based cross-sectional study was conducted at Hawassa University College of Medicine and Health Sciences (HUCMHS) from April 1–30/2021. All randomly selected undergraduate female students at the HUCMHS Campus were the study population.

### 2.2. Sample size determination and sampling technique

The single population proportion formula was used to determine the sample size required to conduct this study from the prevalence of dysmenorrhea occurrence among female students at Mekelle University College of Medicine and Health Science 71.8% (15), at 95% certainty and 5% margin of error to achieve a total sample size of 348 female students after adding 10% of the non-response rate. Out of 720 female students currently studying at CMHS Campus, 348 students were selected by systematic random sampling technique. The departments were listed alphabetically from the Department of Anesthesia to Radiotechnology; then, female students in each department were listed. After obtaining the female student list from the college registrar, it was arranged alphabetically, from anesthesia to radiotechnology; this final list of students was used as the sampling frame. After choosing the first student randomly, it was decided to take every other student with a *K*-value of 2. If there was any absence from class or dropping out, the next student was taken as a sample. Before distributing the questionnaire, any students who were found to be amenorrhoeic, pregnant, within six months post-partum, or currently lactating after being asked were excluded from the study, and the next student was then selected.

#### 2.2.1. Data collection tools and procedures

The data collection tool used in the study was adapted from the literature review and was prepared in English but translated to the local language (Amharic) by two professionals and then again translated back into English by two professionals. Before the questionnaire came into effect, it had been checked for inconsistency. The data collection tools had three parts: sociodemographic characteristics, menstruation characteristics, severity, and the impact of dysmenorrhea on academic activities. The study participants were screened for depression using patient health question nine (PHQ-9) depression screening tools (26, 27). The pain score was made with a numeric pain rating (NPR) and verbal multidimensional pain score system (VMPS) (28). The data collection instrument was pretested on 20 students who were not included in the final analysis, and relevant modifications were instituted before the commencement of actual data collection. Parallelly, data collectors were trained for three days before starting the collecting process. The data was collected by those three trained collectors [trained year two and year three residents (medical doctors on obstetrics and gynecology specialty study)] through a standardized self-administered questionnaire after explaining the questions to those who were unable to understand. The training was given to data collectors by supervisors, including the principal investigator, regarding confidentiality and freedom of the participants to terminate filling out the questionnaire. Supervisors contacted data collectors on a daily basis in case of any problems or difficulties. The completeness of the data was checked by the principal investigator.

#### 2.2.2. Data processing and analysis

Responses for PHQ-9 were summarized based on the mean section total score (by assigning 1 for yes response and 0 for no, or 0–3 for PHQ-9). It was then reported as no depression (0–4), mild depression (5–9), moderate depression (10–14), moderately severe depression (15–19), and severe depression (20–27). Moreover, the presence of anxiety was analyzed using the GAD-7 scale and summarized based on the mean total score (seven questions with a 0–3 rating, and no (0–4), mild (5–9), moderate (10–14), and severe anxiety (15–21)). The severity of pain was assessed using two methods. The first was the NPRS segmented numeric version of the visual analog scale (VAS), where the respondents scored their pain from 0 to 10. The second was the VMPS method, grading the pain severity with analgesia, having systemic symptoms, and affecting daily activities: painful menses requiring analgesics but seldom inhibiting activity (grade I, mild pain); daily activity affected, requiring analgesics, which give sufficient relief (grade II, moderate pain); and daily activity inhibited by dysmenorrhea, with insufficient relief from painkillers (grade III, severe pain). The collected data were checked, cleaned, and entered into Epi data version 7 and exported to SPSS version 21 for further data cleaning and analysis. The information is described in tables, graphs, means, and frequency, including the effect of dysmenorrhea on daily academic activities. The presence of an association between independent and outcome variables was checked by the Pearson chi-square test. Additionally, each independent variable was fitted separately into bivariate logistic analysis to evaluate the degree of association with the outcome variable. Thus, multivariate logistic regression analysis was done on variables with *p*-values less than 0.05. The significance level was obtained at an odds ratio of 95% CI and *p*-value < 0.05.

#### 2.2.3. Ethical clearance and consent

Ethical clearance for written informed consent was obtained from the Institutional Review Board (IRB) of the College of Medicine and Health Sciences, Hawassa University, Ethiopia, with clearance letter no. RPGe/76/2021. The IRB gave ethical clearance. After attaining approval from the institutional review board of Hawassa University, informed consent was taken from the study participants after informing them of the aim of the study. They were informed that confidentiality would be maintained and that they could leave the study at any time if they felt that way inclined. All methods were performed according to the relevant guidelines and regulations.

## 3. Results

### 3.1. Socio-demographic characteristics of students

In total, 99.4% of the respondents were involved in the study. The ages of respondents ranged from 18 to 36 years, with a median of 21 years. The majority, 89.6% (*N* = 310), came from urban areas, whereas 10.4% (*N* = 36) were from rural areas (Table 1).

**Table 1:** Socio-demographic characteristics of students at HU, COMHS, 2021.

Variable		Frequency	Percent	X^2^	*P*-value
Age	Less than 20	12	3.5	6.31	0.091
	20–24	304	87.9		
	25–29	27	7.8		
	Greater than 30	3	.9		
BMI	Less than 18.5	76	22.0	6.173	0.104
	18.5−24.99	246	71.1		
	25−29.99	22	6.4		
	30−39.99	2	.6		
Place of origin	Rural	36	10.4	0.64	0.44
	Urban	310	89.6		
Marital status	Married	30	8.7	14.66	0.001
	Single	316	91.3		
Batch	2nd year	93	26.9	5.5	0.22
	3rd year	117	33.8		
	4th year	65	18.8		
	5th year	33	9.5		
	Medical intern	38	11.0		
Previous pregnancy	Yes	18	5.2	8.2	0.54
	No	328	94.8		
Age of menarche	9–11	20	5.8	2.79	0.44
	12–14	267	77.2		
	15–17	58	16.8		
	Greater than 17	1	.3		
Menses regularity	Regular	271	78.3	0.932	0.334
	Irregular	75	21.7		
Menses interval length	21−35	266	76.9	3.242	0.198
	Greater than 35	21	6.1		
	Irregular	59	17.1		
Duration of menstrual flow	1–3 days	33	9.5	5.3	0.19
	4–5	220	63.6		
	6–7	83	24.0		
	Greater than 7	10	2.9		
Amount of menstrual bleeding	Normal	290	83.8	0.19	0.678
	Excess	56	16.2		
Painful menses (dysmenorrhea)	Yes, during every menses	98	28.3		
	Yes, but intermittently	179	51.7		
	No	69	19.9		

### 3.2. Medical and menstrual history of the study participants

Out of the total 346 respondents, 31 (9%) had a past medical illness, 17 (4.9%) had a past surgical illness, 8 (2.3%) had past abdominopelvic surgery, 16 (4.6%) had a past pelvic infection, and 9 (2.6%) had an ovarian cyst. The history of contraceptive use was reported in 49 (14.2%) participants and pregnancy was reported in 18 (5.2%) participants. The age range for menarche was reported between 9 and 18 years. More than three-quarters of the respondents (77.4%) reported menarche between 12 and 14 years. A large proportion of the study participants reported regular menses (78.3%) (Table 1).

### 3.3. Behavioral activities associated with participants

Out of a total of 346 respondents, 135 (39%) had a variable frequency of sporting activity, 2 (0.6%) smoked cigarettes, 131 (37.9%) had a variable frequency of alcohol consumption, and 66 (19.1%) were sexually active (Table 2).

**Table 2:** Behavioral activities of female students in HU, COMHS, 2021.

Categories	Frequency (346)	Percent
Frequency of sporting activity of female students		
Every day	4	1.2
A few times a week	46	13.3
Once a week	24	6.9
Once a month	8	2.3
Seasonally	25	7.2
Very rarely	38	11.0
I do not exercise	201	58.1
Frequency of alcohol consumption of female students		
A few times a week	12	3.5
Several times a month	4	1.2
During celebrations, parties	56	16.2
Very rarely	59	17.1
Never	215	62.1
Total	346	100.0

### 3.4. Depression, anxiety, and common presentation during menstrual bleeding

Among the students, in general, the most common symptoms associated with menses were back pain (67%); dizziness, weakness, fatigue (65.3%); bloating (55.5%); breast pain (52.9%); and irritability and mood swings (59.8%) (Supplementary Table S1).

**Figure 1:**
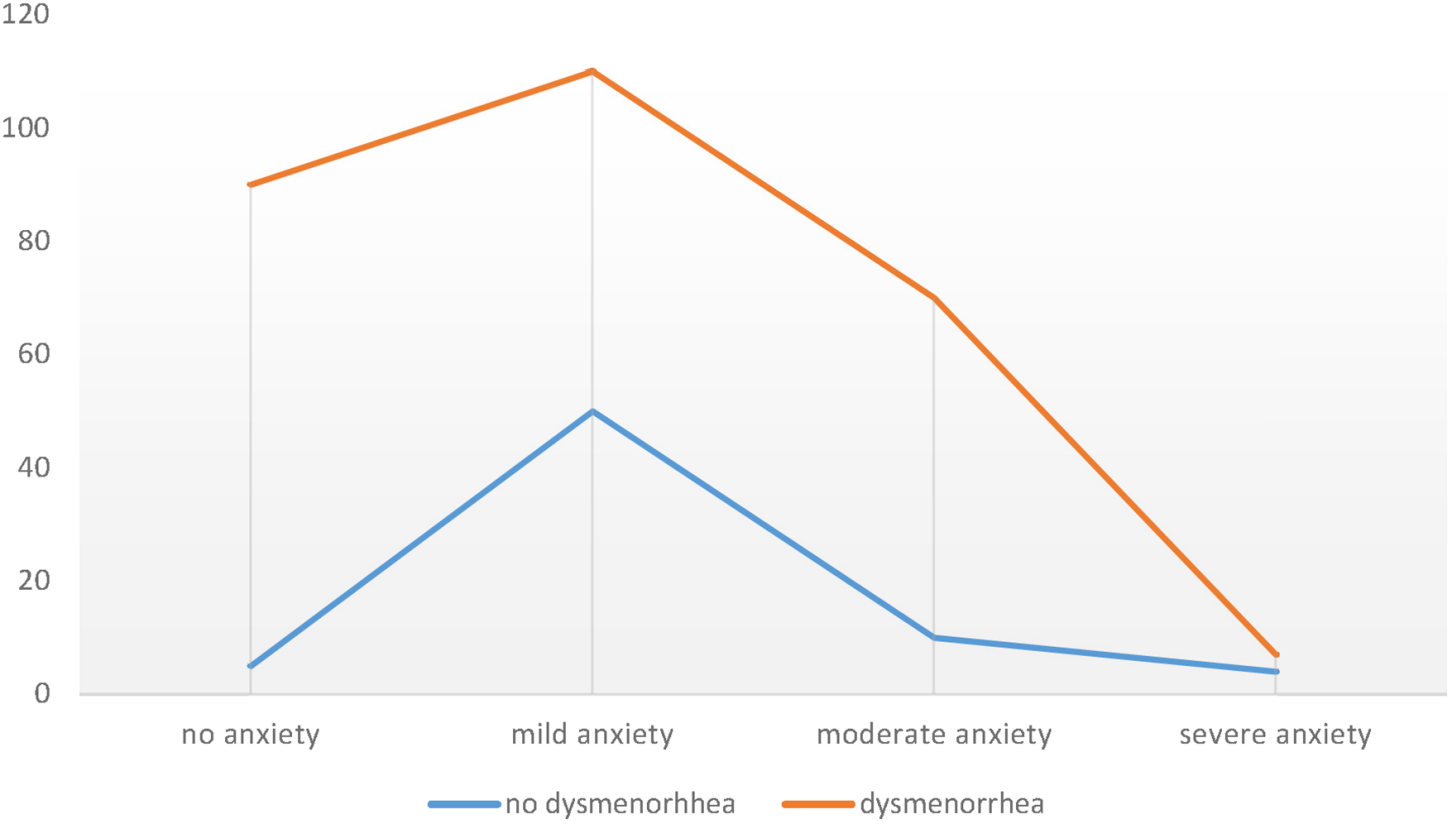
Results of anxiety assessment (GAD-7) of students in HU CMHS in 2021.

**Figure 2:**
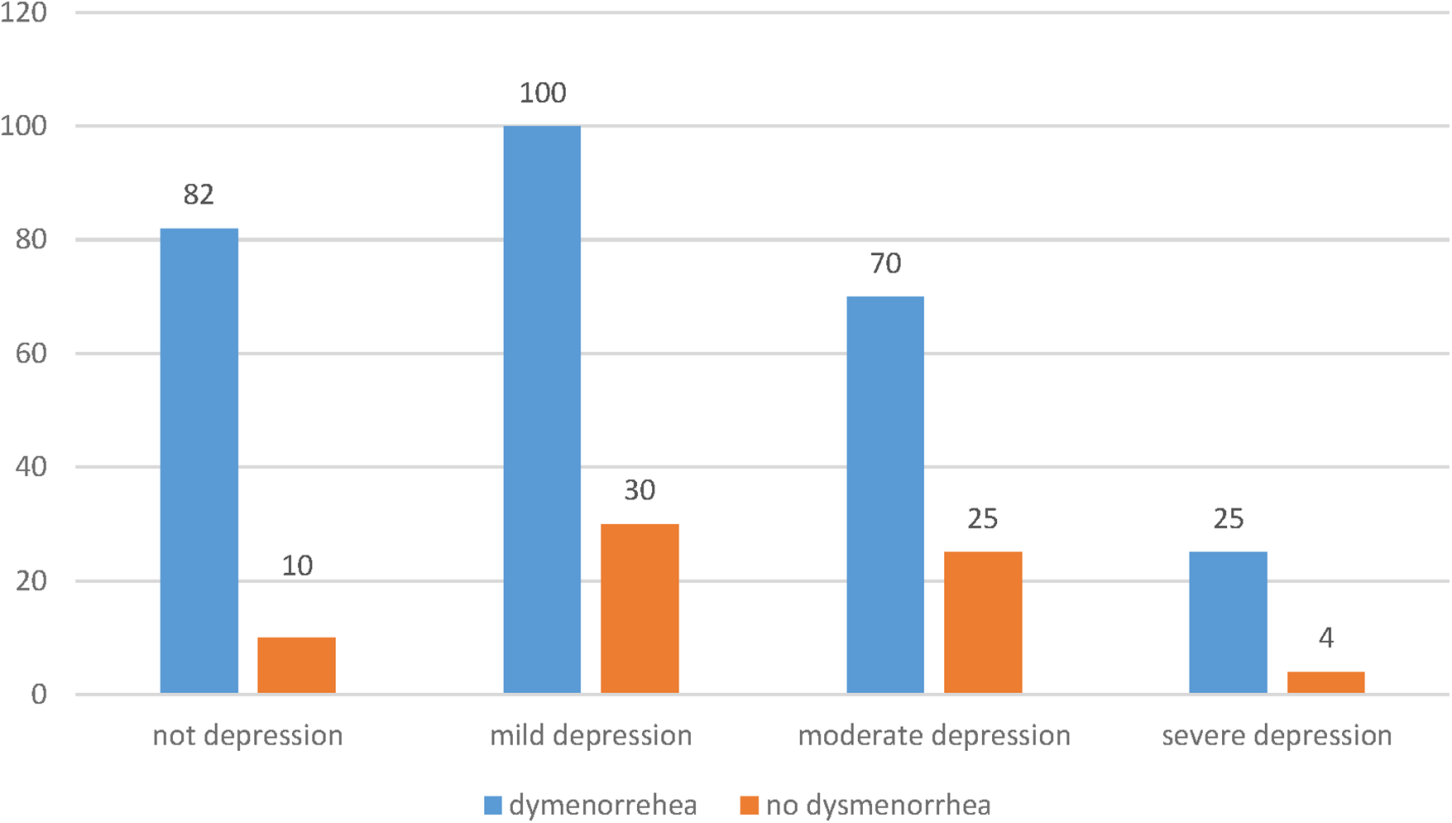
Result of positive screening of depression (PHQ-9) assessment of students in HU, CMHS in 2021.

Of all students, 72% reported anxiety and 73% had positive screening for depression. Moreover, 74% of students who reported anxiety (Figure 1) and 76% of those with positive screening for depression (Figure 2) had complained of dysmenorrhea.

### 3.5. Prevalence of dysmenorrhea and its effect on students' academic performance

The prevalence of dysmenorrhea was found to be 80.1% (Table 3). Using the Numeric Pain Rating Scale, out of 277 students with dysmenorrhea, 21.7% (60), 33.2% (92), 37.5% (104), and 7.6% (21) of the respondents reported mild, moderate, severe, and very severe intensity of pain, respectively. According to the verbal multidimensional scoring system, out of 277 students, 47.6% (132) of students' menstruation was painful but seldom inhibited normal activity, seldom requiring analgesics (mild pain). In total, 39.7% (110) of students' daily activity was affected; analgesics were required and gave sufficient relief so that absence from school was unusual (moderate pain). In 12.6% (35) of students, daily activity was inhibited by dysmenorrhea, with analgesics giving insufficient pain relief (severe pain).

**Table 3:** Dysmenorrhea prevalence and severity, Hawassa University, COMHS, 2021.

Variable		Frequency	Percent (%)
Painful menses (dysmenorrhea)	No	69	19.9
	Yes	277	80.1
Dysmenorrhea occurs	During every menses	98	35.4
	Yes, but intermittently	179	64.6
When did dysmenorrhea start relative to menarche	Started with my menarche	73	26.4
	One year after the onset of menarche	59	21.3
	2–5 years after the onset of menarche	90	32.5
	>5 years after menarche	55	19.9
When does the pain start relative to menses flow	A day before menses	85	30.7
	Day 1 of menses	176	63.5
	The second or third day of menses	16	5.8
When does the pain reach a peak	A day before menses	30	10.8
	Day 1 of menses	202	72.9
	The second or third day of menses	41	14.8
	After cessation of menses	4	1.4
Duration of painful menses	Less than a full day	15	5.4
	1–2 days	210	75.8
	3–4 days	42	15.2
	Greater than four days	10	3.6
Treated for dysmenorrhea	Yes	42	15.2
	No	235	84.8
Numeric pain scaling	Mild 0–3	60	21.7
	Moderate 4–6	92	33.2
	Severe 7–9	104	37.5
	Very severe 10	21	7.6
Severity grading	Grade 1 (mild)	132	47.7
	Grade 2 (moderate)	110	39.7
	Grade 3 (severe)	35	12.6

#### 3.5.1. The impact of dysmenorrhea on academic performance

Among the students with dysmenorrhea, the most common symptoms associated were back pain (71.8%); dizziness, weakness, and fatigue (75.1%); bloating (62.8%); and breast pain (58.1%). Of the respondents with dysmenorrhea, 83% (194) reported that the disorder interfered with their academic performance; of these, 48% were mildly affected, 25.6% were moderately affected, and 9% were severely affected. Dysmenorrhea interfered with the concentration of 86.6% (*N* = 240) of participants; of these, 46.2% were mildly affected, 26.7% were moderately affected, and 13.7% were severely affected (Table 4).

**Table 4:** Effects of dysmenorrhea on academic activities among dysmenorrhea and based on the grade of pain severity, at HU, COMHS, 2021.

Variable	Categories	Dysmenorrhea	Grade I pain	Grade II pain	Grade III pain
		Frequency (Percent)	Frequency (Percent)	Frequency (Percent)	Frequency (Percent)
Academic performance	Not affected	47 (17.0)	43 (32.6)	4 (3.6)	0
	Mildly affected	134 (48.4)	74 (56.1)	52 (47.3)	8 (22.9)
	Moderately affected	71 (25.6)	12 (9.1)	51 (46.4)	8 (22.9)
	Severely affected	25 (9.0)	3 (2.3)	3 (2.7)	19 (54.3)
Concentration	Not affected	37 (13.4)	31 (23.5)	6 (5.5)	0
	Mildly affected	128 (46.2)	75 (56.8)	49 (44.5)	4 (11.s4)
	Moderately affected	74 (26.7)	20 (15.2)	45 (40.9)	9 (25.7)
	Severely affected	38 (13.7)	6 (4.5)	10 (9.1)	22 (62.9)
Study	Not affected	36 (13.0)	31 (23.5)	4 (3.6)	1 (2.9)
	Mildly affected	108 (39.0)	72 (54.5)	34 (30.9)	2 (5.7)
	Moderately affected	86 (31.0)	21 (15.9)	55 (50.0)	10 (28.6)
	Severely affected	47 (17.0)	8 (6.1)	17 (15.5)	22 (62.9)
Sleep	Not affected	76 (27.4)	57 (43.2)	15 (13.6)	4 (11.4)
	Mildly affected	93 (33.6)	45 (34.1)	44 (40.0)	4 (11.4)
	Moderately affected	76 (27.4)	24 (18.2)	43 (39.1)	9 (25.7)
	Severely affected	11.6 (32)	6 (4.5)	8 (7.3)	18 (51.4)
Personal relationship	Not affected	76 (27.4)	56 (42.4)	19 (17.3)	1 (2.9)
	Mildly affected	109 (39.4)	58 (43.9)	46 (41.8)	5 (14.3)
	Moderately affected	71 (25.6)	15 (11.4)	41 (37.3)	15 (42.9)
	Severely affected	21 (7.6)	3 (2.3)	4 (3.6)	14 (40.0)
Class absence (days)	Yes	49.1	36 (27.3)	71 (64.5)	29 (82.9)
	No	50.9	96 (72.7)	39 (35.5)	6 (17.1)
Class absence (days)	0 day	141 (50.9)	96 (72.7)	39 (35.5)	6 (17.1)
	1 day	101 (36.5)	29 (22.0)	56 (50.9)	16 (45.7)
	2 days	30 (10.8)	6 (4.5)	15 (13.6)	9 (25.7)
	3 days	5 (1.8)	1 (0.8)	0	4 (11.4)
Reason for absence	Lack of concentration	17 (12.5)	9 (25)	6 (8.5)	2 (6.9)
	Pain intensity	109 (80.1)	20 (55.6)	63 (88.7)	26 (89.7)
	Lack of interest	10 (7.4)	7 (19.4)	2 (2.8)	1 (3.5)
	Total	277	132 (100.0)	110 (100.0)	35 (100)

### 3.6. The impact of dysmenorrhea on academic activities and factors associated with dysmenorrhea

The potential impact of dysmenorrhea on academic activities was sought through chi-square analysis, and it was found to have a negative impact on academic performance (*p* = 0.00), concentration (*p* = 0.00), study time (*p* = 0.00), relationships (*p* = 0.00), and sleeping time (*p* = 0.00) (Table 5). Furthermore, in bivariate logistic analysis, the following factors were identified as significantly associated with dysmenorrhea: marital status, use of contraception, pregnancy, sexual activity, maternal dysmenorrhea, sister with a history of dysmenorrhea, and clot in menses. However, multivariate logistic regression analysis identified the following factors as being significantly associated with dysmenorrhea: being sexually active, maternal history of dysmenorrhea, sister with a history of dysmenorrhea, and presence of depression.

**Table 5:** The association of academic performance with dysmenorrhea using Chi-square, students at HU, COMHS 2021.

Variables		Dysmenorrhea		X^2^	*P*-value
		Yes	No		
		*N* (%)	*N* (%)		
Academic performance affected	Yes	230 (93.5)	16 (6.5)	96.8	0.00
	No	47 (47)	53 (53)		
Relationship affected	Yes	201 (95.7)	9 (4.3)	82.9	0.00
	No	76 (55.9)	60 (44.1)		
Concentration affected	Yes	240 (93.4)	17 (6.6)	111.1	0.00
	No	37 (41.6)	52 (58.4)		
Study time affected	Yes	241 (93.1)	18 (6.9)	108.9	0.00
	No	36 (41.4)	51 (58.6)		
Sleeping affected	Yes	201 (95.3)	10 (4.7)	78.2	0.00
	No	76 (56.3)	59 (43.7)		

Being sexually active was the determinant factor of dysmenorrhea. Sexually active female students were more than three times more likely to develop dysmenorrhea compared to those who were not sexually active [AOR = 3.260 (1.00–10.647)]. Family history is a strong risk factor for dysmenorrhea: for those with a mother and sister with dysmenorrhea, the risk was AOR = 8.481 (95% CI, 3.608–19.934) and AOR = 4.012 (95% CI, 1.929–8.346) times higher than those without a family history of dysmenorrhea, respectively. Students who had a history of positive screening for depression had a 5.7 times higher risk compared to those who had no history of depression (AOR = 5.683, (95% CI, 1.762–18.329) (Table 6; Supplementary Table S2).

**Table 6:** Shows the results of the multivariate analysis using significant bivariate variables, of students at HU, COMHS 2021.

Variables		COR (95% CI)	AOR (95% CI)	*P*-value
Married	Yes	0.241 (0.111–0.522)	1.4 (0.99–4.32)	0.45
	No	1	1	
Sexually active	Yes	0.335 (0.185–0.607)	0.307 (0.094–1.001)	0.03
	No	1	1	
Using contraception	Yes	0.448 (0.230–0.873)	1.154 (0.243–5.4)	0.894
	No	1	1	
Pregnancy history	Yes	0.175 (0.066–0.464)	0.594 (0.23–5.94)	0.641
	No	1		
Maternal has dysmenorrhea	Yes	9.374 (4.597–19.115)	8.481 (3.608–19.934)	0.00
	No	1	1	
Sister has dysmenorrhea	Yes	5.261 (2.978–9.294)	4.012 (1.929–8.346)	0.00
	No	1	1	
Chewing khat	Yes	0.299 (0.078–1.143)	1.289 (1.09–2.89)	0.56
	No	1	1	
Alcohol consumption	Yes	1.506 (0.854–2.653)	1.68 (2.04–4.34)	0.32
	No	1	1	
clot presence in menses	Yes	2.628 (1.199–5.761)	3.95 (2.45–8.09)	0.70
	No	1	1	
Having anxiety and worry	Yes	6.160 (2.396–15.837)	5.83 (3.90–5.72)	0.71
	No	1		
Positive screening for depression	Yes	2.481 (1.210–5.088)	5.683 (1.762–18.329)	0.041
	No	1	1	
Irritable and mood Swings	Yes	2.480 (1.449–4.245)	7.23 (2.003–4.712)	0.54
	No	1	1	
Age	≤20	1	1	
	21–24	0.167 (0.11–2.56)	1.34 (1.10–3.08)	0.49
	25–29	0.113 (0.10–1.34)	2.36 (0.45–2.612)	0.51
	≥30	0.211 (0.17–167)	1.02 (0.742–1.83)	0.62
BMI	<18.5	1	1	
	18.5−24.99	0.152 (0.009–2.62)	0.53 (0.98–3.89)	0.89
	25−29.99	0.295 (0.018–2.98)	0.739 (1.23–3.78)	0.71
	30−39.99	0.100 (0.004–2.287	1.43 (0.87–2.07)	0.81
Batch	2nd year	1	1	
	3rd year	2.56 (0.902–7.25)	5.6 (1.52–4.19)	0.31
	4th year	1.36 (0.49–3.95)	4.9 (1.034–2.91)	0.98
	5th year	1.494 (0.485–4.63)	2.91 (2.71–3.49)	0.67
	Medical intern	1.46 (0.47–6.35)	3.1 (2.08–3.12)	0.71
Residency	Rural	1.389 (0.631–3.12)	2.81 (0.32–1.98)	0.68
	Urban	1	1	
Age at menarche	9–11	1	1	
	12–14	13 (0.123–11.56)	2.1 (3.41–8.31)	0.32
	15–17	2.45 (0.98–9.13)	4.2 (0.34–2.40)	0.51
	Greater than 17	4.45 (0.56–5.34)	3.21 (1.6–3.13)	0.12
Menses interval length	21−35	1	1	
	≥35	1.045 (0.519–2.101)	2.15 (0.39–1.57)	0.81
	Irregular	0.196 (0.24–2.67)	9 (2.13–3.91)	0.39
Duration of menstrual flow	1–3 days	1	1	
	4–5	2.4 (0.261–22.45)	3.6 (1.35–2.09)	0.31
	6–7	2.76 (0.336–21.97)	4.1 (0.98–1.32)	0.92
	≥7	1.233 (0.141–10.78)	7.1 (0.73–3.29)	0.48
Amount of menstrual bleeding	Normal	1	1	
	Excess	1.175 (0.56–2.56)	1.02 (0.49–3.15)	0.41
Tea	No	1	1	
	1 cup	0.15 (0.11–1.45)	1.2 (0.34–5.12)	0.49
	2 cups	0.127 (0.11–176)	0.87 (0.91–5.86)	0.71
	3 cups	0.115 (0.10–1.4)	0.42 (0.37–9.41)	0.59
	4 cups	0.05 (0.01–1.117)	2.1 (0.712–8.71)	0.61

AOR, adjusted odds ratio; COR, crude odds ratio.

## 4. Discussion

This study shows a prevalence of dysmenorrhea of 80.1%, which is comparable to studies done in northern Ethiopia, Nigeria, and Morocco (2, 12, 16) and other systemic reviews and meta-analysis (7–10), whereas the prevalence found in this study is higher than those of the study done in eastern Ethiopia, 69.3% (29); Mekelle University, 71.8% (15); and Kenya, 68.1% (23). The prevalence found in this study is lower than that found in Kuwait University (30), Lithuania Vilnius University (19), and Turkey (1). The variation is due to the assessment tool, method of data collection, sociocultural, ethnic, and lifestyle factors among females, as well as the absence of a universally accepted definition. Moreover, menstruation is considered a private issue in many cultures, so its associated complaints might be kept silent by most females. Above all, pain tolerance and reporting have been influenced by the cultural, religious, and traditional practices from one geographical area to the other. There are different coping mechanisms for pain across cultures and geography. Furthermore, the study population was comprised of university students who were away from their families, so there was no one to take care of them during painful menses, which made the threshold for pain low in this study population (30).

In this study, the prevalence of dysmenorrhea was found to be 80.1% (277) using the Numeric Pain Rating Scale; of those affected by dysmenorrhea, 21.7% (60), 33.2% (92), 37.5% (104), and 7.6% (26) reported mild, moderate, severe, and very severe pain intensity, respectively. This is similar to the findings of one study where 47.4% of unmarried women reported severe and very severe pain (31). According to the verbal multidimensional scoring system, 132 (38.1%) students had grade I mild pain, and 110 (32.1%) females had grade II moderate pain. Additionally, in 10.1% (35) of students, daily activity was inhibited by dysmenorrhea, and the effect of analgesics on the pain was insignificant (grade III, severe pain). This matches the findings of a study in Ethiopia and an Indian study where 55.26% of unmarried women were found to use pain relief on the second day of their menses) (16, 31). It is slightly higher than the systemic reviews done in 2022, wherein 31.1%, 25.7%, and 8.3% of girls were found to have mild, moderate, and severe pain, respectively (10). So, irrespective of the pain scoring methods, almost half of the female students suffered severe pain during menstruation, which shows how profound and complicated the problem is.

Of the respondents with dysmenorrhea, 83% reported that the disorder interfered with their academic performance, which was in line with the findings in northern Ethiopia and slightly lower than the study in Debre Brehan (15, 16) but higher than findings in Ghana and Turkey (1, 22). This may be due to socio-cultural differences, differences in pain tolerance between the populations, and methodological differences. Additionally, this might be due to the questionnaire design used to identify the effect on academic activities: ours included options (not affected, mildly affected, moderately affected, and severely affected), whereas the previously mentioned Turkey and Ghana studies used Yes/No options, whereby the students with mild negative effects might have chosen No in the Yes/No options.

In our study, 38% of all women reported interfering with their regular daily activities. Our finding was higher than studies done in Dutch which is one of the largest studies done so far to look into the impact of dysmenorrhea. The discrepancy may be due to the difference in the source population that is a community study (12). It interfered with the concentration of 86.6% of students, which was higher than the findings in Debre Brehan, Ghana, and Australia (16, 23, 32). More than two-thirds reported dysmenorrhea had interfered with their relationship (mild in 39.4% and moderate to severe in 33.2%), which was higher than in Debre Brehan, Mekelle, Ghana, and Australia (15, 16, 23, 32). It was lower than in Turkey, where 92% reported personal relationships were negatively affected by dysmenorrhea (1). This might be due to the questionnaire design used to identify effects on academic activities and differences in the study population. Additionally, there are different pain coping mechanisms across the regions and cultures. Therefore, due to dysmenorrhea, personal relationships and concentration are significantly affected.

Almost half of the students with dysmenorrhea, 49.1%, were found to have been absent from class during menses for 1–2 days. The most common reason for class absence was pain intensity, reported at 80.1%. This finding is higher than those of the studies in Mekelle, Ghana, and Australia (15, 23, 32) and lower than those of the studies in Debre Brehan and Gondar (14, 16). This may be due to differences in geography, culture, and pain tolerance. The study population was not similar across the studies: some included women in the community (33) and some included high school students (14), whereas our study used university students.

As indicated in this study, menstrual disturbances like dysmenorrhea and premenstrual syndrome have a significant negative impact on the quality of life of students. It affects the productivity of students through an increased rate of school absenteeism, poor academic performance, and poor social relationships with friends and families. Menstrual flow is controlled by the hypothalamus-pituitary gonadal/adrenal axis (HPG/A). The HPG axis regulates and controls it through different feedback mechanisms so that hormonal changes during menses are in strict hemostasis. It has been reported that different lifestyle factors, such as psychological, emotional, or physical stress, can negatively affect the HPG axis, thereby resulting in menstrual disturbances. Stressful conditions result in elevated levels of cortisol and energy demand, which can disrupt the normal hormonal changes during menses by affecting the HPG axis (9).

Being sexually active was the determinant factor of dysmenorrhea. Sexually active female students were found to be 70% less likely to develop dysmenorrhea than those who were not. This finding is similar to that of multiple studies across the scientific world (14, 15, 17, 24) and may be due to sexually active women being more likely to use contraceptive options, especially hormonal ones, which are protective against dysmenorrhea. Since the pathophysiology of dysmenorrhea is primarily hormonal disturbance around premenstrual time, hormonal contraceptives have a protective effect and are used as a treatment for dysmenorrhea.

Students whose mother and sister had a history of dysmenorrhea were found to be eight and four times more likely to experience dysmenorrhea, respectively, than students whose mother and sister had not experienced dysmenorrhea. Most studies revealed that females who had a positive family history of dysmenorrhea were more likely to develop the condition (14, 15, 22, 21). This study showed the presence of positive screening for depression was significantly associated with dysmenorrhea. Participants who had screened positive for depression had a 5.7 times higher risk of dysmenorrhea than those who had not. Mood disorders were associated with dysmenorrhea as this group has hypersensitivity to pain (17). Having depression was associated with a higher risk of dysmenorrhea (16). Furthermore, the strong effect of being depressed is associated with the risk of menstrual pain, with an odds ratio of 13.3 (33). There is no well-established mechanism for depression to increase the risk of dysmenorrhea; however, there is an overlapping neurohormonal and neurochemical mechanism in the pathophysiology of dysmenorrhea and depression. The progesterone metabolite will bind to the neuro-steroid binding site of the GABA (gamma-aminobutyric acid) receptor, rendering it resistant to activation; it will then decrease centrally GABA-mediated inhibition. Additionally, a lowering of serotonin levels is observed, and the serotonergic functioning pathway is estimated to be deficient in the brain by estimating its activity, so medications that augment serotonin are effective. However, the above mechanisms are associated with premenstrual syndromes, not merely dysmenorrhea (34).

Finally, it is recommended to provide counseling and health education to young female students regarding coping mechanisms for dysmenorrhea and pharmacologic management with nonsteroidal anti-inflammatory drugs and hormonal contraceptives, which are effective interventions (5, 6).

## 5. Strengths

This study aimed to assess the factors and impacts of dysmenorrhea in young females. Moreover, it used two different scoring methods, especially numerical pain scoring, which has not been used widely in the literature.

## 6. Limitations of the study

Other possible causes of secondary dysmenorrhea, like endometriosis, PCOS (polycystic ovarian syndrome), and so on, were not excluded since they require investigation. The cross-sectional study lacks cause and effect association.

## 7. Conclusion

The prevalence of dysmenorrhea is relatively high among the female students attending university included in this study. It has a significant negative impact on students' academic performance. This study showed that being sexually active is protective against dysmenorrhea. Maternal history of dysmenorrhea, having a sister with a history of dysmenorrhea, and the presence of depression were significantly associated with dysmenorrhea.

## Data Availability

The original contributions presented in the study are included in the article/**Supplementary Material**, further inquiries can be directed to the corresponding author.
