# Supplementary material for: Prevalence of dysmenorrhea and associated factors and its effect on daily academic activities among female undergraduate students of Hawassa University College of Medicine and Health Sciences, Hawassa, Ethiopia
Source: Front Reprod Health. 2023 Dec 15;5:1244540. doi: 10.3389/frph.2023.1244540 (PMC10757849; doi:10.3389/frph.2023.1244540)
Supplement: Supplementary file 1 [file Table1.docx]

**Supplementary Table 1**. symptoms associated with menses, among dysmenorrhic students at HU COMHS, 2021

| Variables | Categories | Frequency | Percent (%) |
| --- | --- | --- | --- |
| Nausea | Yes | 118 | 42.6 |
|  | No | 159 | 57.4 |
| Vomiting | Yes | 66 | 23.8 |
|  | No | 211 | 76.2 |
| Dizziness, weakness, fatigue | Yes | 208 | 75.1 |
|  | No | 69 | 24.9 |
| Breast pain | Yes | 161 | 58.1 |
|  | No | 116 | 41.9 |
| Bloating | Yes | 174 | 62.8 |
|  | No | 103 | 37.2 |
| Leg cramp | Yes | 125 | 45.1 |
|  | No | 152 | 54.9 |
| Back pain | Yes | 199 | 71.8 |
|  | Yes | 78 | 28.2 |
| Anxiety and worries | Yes | 90 | 32.5 |
|  | No | 187 | 67.5 |
| Irritability and mood swinging | Yes | 178 | 64.3 |
|  | No | 99 | 35.7 |
| Depression | Yes | 62 | 22.4 |
|  | No | 215 | 77.6 |
| Premenstrual syndrome | Yes | 129 | 46.6 |
|  | No | 148 | 53.4 |

| V**ariables** |  | **Dysmenorrhea** | | **X^2^** | **P-value** |
| --- | --- | --- | --- | --- | --- |
|  |  | **Yes** | **No** |  |  |
|  |  | **N (%)** | **N (%)** |  |  |
| Married | Yes | 16(53.3) | 14(46.7) | 14.66 | 0.001 |
|  | No | 261(82.6) | 55(13.4) |  |  |
| Sexually active | Yes | 42(39.4) | 24(60.6) | 13.7 | 0.001 |
|  | No | 235(83.4) | 45(16.6) |  |  |
| Using contraception | Yes | 33(67.3) | 16(32.7) | 5.66 | 0.016 |
|  | No | 244(82.2) | 53(17.8) |  |  |
| Pregnancy history | Yes | 8(44.4) | 10(55.6) |  |  |
|  | No | 269(82) | 59(18) |  |  |
| Maternal has dysmenorrhea | Yes | 170(94.4) | 10(5.6) | 48.6 | 0.0001 |
|  | No | 107(64.4) | 59(35.5) |  |  |
| Sister has dysmenorrhea | Yes | 197(90) | 22(10) | 36.602 | 0.000 |
|  | No | 80(63) | 47 (37) |  |  |
| Chewing chat | Yes | 5(55.6) | 4(44.4) | 3.475 | 0.062 |
|  | No | 272(80.7) | 65(19.3) |  |  |
| Alcohol drink | Yes | 167(77.7) | 48(22.3) | 2.2 | 0.15 |
|  | No | 110(84) | 21(16) |  |  |
| clot presence in menses | Yes | 71(89.9) | 8(10.1) | 6.178 | 0.013 |
|  | No | 206(77.2) | 61(28.2) |  |  |
| Having anxiety and worry | Yes | 187(74.5) | 64(25.5) | 17.64 | 0.0001 |
|  | No | 90(94.7) | 5(5.3) |  |  |
| Presence depression | Yes | 195(76.8) | 59(23.2) | 6.46 | 0.011 |
|  | No | 82(89.1) | 10(10.9) |  |  |
| Irritable Mood Swinging | Yes | 178(86) | 29 (14) | 11.359 | 0.01 |
|  | No | 99(71.2) | 40 (28.8) |  |  |
| Regularity of menstrual cycle | irregular | 63 (84) | 12(16) | 0.932 | 0.334 |
|  | Regular | 214((78.9) | 57(11.1) |  |  |
| Age | less than 20 | 9 | 3 | 6.32 | 0.091 |
|  | 20-24 | 248 | 56 |  |  |
|  | 25 – 29 | 19 | 8 |  |  |
|  | greater than 30 | 1 | 2 |  |  |
| BMI | less than 18.5 | 66 | 10 | 6.173 | 0.104 |
|  | 18.5 -24.99 | 190 | 56 |  |  |
|  | 25 -29.99 | 20 | 2 |  |  |
|  | 30 -34.99 | 1 | 1 |  |  |
| residency | Rural | 27 | 9 | 0.64 | 0.44 |
|  | Urban | 250 | 60 |  |  |
| Batch | 2nd year | 67 | 26 | 5.5 | 0.22 |
|  | 3rd year | 97 | 20 |  |  |
|  | 4th year | 53 | 12 |  |  |
|  | 5th year | 27 | 6 |  |  |
|  | medical intern | 33 | 5 |  |  |
| Age of menarche | 9-11 | 16 | 4 | 2.79 | 0.44 |
|  | 12-14 | 218 | 49 |  |  |
|  | 15-17 | 42 | 16 |  |  |
|  | greater than 17 | 1 | 0 |  |  |
| Menses interval length | 21 -35 | 210(78.9) | 56(21.1) | 3.242 | 0.198 |
|  | greater than 35 | 20(95.2) | 1(4.8) |  |  |
|  | Irregular | 47(79.7) | 12(20.3) |  |  |
| Duration of menstrual flow | 1-3 days | 26(78.8) | 7(21.2) | 5.3 | 0.19 |
|  | 4-5 | 169(76.8) | 51(23.2) |  |  |
|  | 6-7 | 73(88) | 10(12) |  |  |
|  | greater than 7 | 9(90) | 1(10) |  |  |
| Amount of menstrual bleeding | Normal | 231(79.1) | 59(20.1) | 0.19 | 0.678 |
|  | Excess | 46(82.1) | 10(17.9) |  |  |
| Tea | No | 64(80) | 16(20) |  |  |
|  | 1 cup | 15479.8) | 39(20.2) |  |  |
|  | 2 cups | 48((81.4) | 11(18.6) |  |  |
|  | 3 cups | 10(90.9) | 1(9.1) |  |  |
|  | 4 cups | 1(33.3) | 2(66.7) |  |  |

**Supplementary Table 2:** Shows summary of variables and their chi square of students at HUCOMHS 2021
